# Vesicle-based cell-free synthesis of short and long unspecific peroxygenases

**DOI:** 10.3389/fbioe.2022.964396

**Published:** 2022-11-01

**Authors:** Ruben Magnus Walter, Anne Zemella, Marina Schramm, Jan Kiebist, Stefan Kubick

**Affiliations:** ^1^ Fraunhofer Institute for Cell Therapy and Immunology (IZI), Branch Bioanalytics and Bioprocesses (IZI-BB), Potsdam, Germany; ^2^ Institute of Biotechnology, Brandenburg University of Technology Cottbus-Senftenberg, Senftenberg, Germany; ^3^ Freie Universität Berlin, Institute of Chemistry and Biochemistry – Biochemistry, Berlin, Germany; ^4^ Faculty of Health Sciences, Joint Faculty of the Brandenburg University of Technology Cottbus – Senftenberg, The Brandenburg Medical School Theodor Fontane, University of Potsdam, Potsdam, Germany

**Keywords:** cell-free protein synthesis, *in vitro* transcription/translation, enzymes, unspecific peroxygenases, insect cell lysate

## Abstract

Unspecific peroxygenases (UPOs, EC 1.11.2.1) are fungal enzymes that catalyze the oxyfunctionalization of non-activated hydrocarbons, making them valuable biocatalysts. Despite the increasing interest in UPOs that has led to the identification of thousands of putative UPO genes, only a few of these have been successfully expressed and characterized. There is currently no universal expression system in place to explore their full potential. Cell-free protein synthesis has proven to be a sophisticated technique for the synthesis of difficult-to-express proteins. In this work, we aimed to establish an insect-based cell-free protein synthesis (CFPS) platform to produce UPOs. CFPS relies on translationally active cell lysates rather than living cells. The system parameters can thus be directly manipulated without having to account for cell viability, thereby making it highly adaptable. The insect-based lysate contains translocationally active, ER-derived vesicles, called microsomes. These microsomes have been shown to allow efficient translocation of proteins into their lumen, promoting post-translational modifications such as disulfide bridge formation and N-glycosylations. In this study the ability of a redox optimized, vesicle-based, eukaryotic CFPS system to synthesize functional UPOs was explored. The influence of different reaction parameters as well as the influence of translocation on enzyme activity was evaluated for a short UPO from *Marasmius rotula* and a long UPO from *Agrocybe aegerita*. The capability of the CFPS system described here was demonstrated by the successful synthesis of a novel UPO from *Podospora anserina*, thus qualifying CFPS as a promising tool for the identification and evaluation of novel UPOs and variants thereof.

## 1 Introduction

Unspecific peroxygenases (UPO, EC 1.11.2.1) are a recently discovered group of heme-containing oxidoreductases. Initially identified as a haloperoxidase in 2004, *Aae*UPO from *Agrocybe aegerita* (syn. *Cyclocybe aegerita*) was the first UPO described. It is a secreted glycoprotein with oxidizing activity towards several substrates (Ullrich et al., 2004). In the following years, UPOs were found to be secreted by other fungi as well (Anh et al., 2007; Gröbe et al., 2011), and further characterization revealed a broad catalytic spectrum with respect to both the substrate range and the numerous catalyzed reactions. Their catalytic repertoire includes one-electron oxidations (as for a classical peroxidase) as well as the activation of C-H (Peter et al., 2011; Babot et al., 2013) and C=C bonds (Peter et al., 2013), the cleavage of ethers (Kinne et al., 2009), and the oxygenation of heteroatoms (Ullrich 2009). The substrates of UPOs ranges from small aromatic (Kluge et al., 2009) or aliphatic (Babot et al., 2013) molecules to more sterically demanding substrates such as steroids (Babot et al., 2015). An overview of their catalytic diversity is given by Hofrichter et al. (Hofrichter et al., 2022). Because of this versatility, UPOs are promising candidates for the development of novel biosynthetic pathways. In this context, UPOs were successfully used to produce isophorone derivatives that are important precursors for the synthesis of pharmaceuticals (Aranda et al., 2019) and generate building blocks from renewable sources such as 5-hydroxymethylfurfural for the polymer industry (Carro et al., 2014). The ability of UPOs to generate pharmacologically relevant substrates is also of particular interest. The conversion of the antiplatelet drug clopidogrel to its active metabolite by different UPOs was also recently demonstrated (Kiebist et al., 2021).

Despite their vast capabilities, the full potential of UPOs remains untapped partly because of their limited availability. Through the extensive screening of genomic data, over 4,000 putative UPO genes have been identified and these can be roughly divided into long and short UPOs (Kinner et al., 2021). However, until today, only a little over 20 different UPOs have been expressed in an active form (Hofrichter et al., 2022). Two main strategies have been applied: homologous expression in the wild-type host and heterologous expression, mostly in Pichia pastoris (syn. *Komagataella phaffii*). The cultivation of wild-type UPO producers sometimes results in large amounts of a single UPO as reported for *Marasmius rotula* UPO (*Mro*UPO) (Gröbe et al., 2011) yielding 445 mg/L. In addition to the time-consuming cultivation, a major drawback of producing UPOs in their original host is often the poor accessibility of the host to genetic engineering, which limits the production of UPOs to the unmodified wild-type enzyme. However, genetically engineered UPOs can outperform their wild-type equivalent in terms of secretion levels, catalytic activity (Molina-Espeja et al., 2014), substrate specificity (Gomez de Santos et al., 2019), and solvent stability (Martin-Diaz et al., 2021). In order to overcome these limitations, several classical production hosts have been examined for UPO expression. In principle, recombinant expression in *E*. *coli* is possible but typically leads to low amounts of active enzyme (Carro et al., 2019; Linde et al., 2020). The use of fungal production hosts such as *Saccharomyces cerevisiae* (Molina-Espeja et al., 2015) and *Aspergillus oryzae* (Babot et al., 2013) have proven to be more promising. In this context, *P. pastoris* has to be highlighted. In recent years, considerable progress has been made in establishing *P. pastoris* as a heterologous host for UPO production. The production of 735 g recombinant *Aae*UPO in a 2500 L fermentation was recently reported (Tonin et al., 2021). Several other UPOs, including newly characterized UPOs, were also produced in *P. pastoris* (Püllmann and Weissenborn, 2020; Bormann et al., 2021; Püllmann et al., 2021; Rotilio et al., 2021). However, not all UPOs can be easily produced in *P. pastoris* as recently demonstrated by Bormann et al. wherein only three out of 10 UPOs were expressed in an active form (Bormann et al., 2021). To improve UPO production in *P. pastoris*, extensive efforts were made to identify favorable promotors and signal peptides, which would allow an efficient UPO expression and secretion (Püllmann and Weissenborn 2020; Püllmann et al., 2021). Although these studies have shown promising results, including the successful expression of novel UPOs, there were still UPOs that could not be expressed in an active form such as *Gma*UPO and *Mwe*UPO (Püllmann et al., 2021), that can be expressed by and isolated from only its natural host *Marasmius wettseinii* (Ullrich et al., 2018). A universal platform for UPO production is thus yet to be shown.

A cell-free approach for the production of recombinant UPOs was recently published (Schramm et al., 2022). In a cell-free protein synthesis (CFPS), translationally active cell-lysate is used to carry out translation reactions. Because of its open nature, reaction conditions can be directly manipulated, thus making it a versatile tool for protein production (Gregorio et al., 2019). CFPS has proven valuable for the synthesis of numerous proteins (Gregorio et al., 2019; Dondapati et al., 2020), including heme-containing proteins such as horseradish peroxidase (HRP) (Zhu et al., 2015; Park and Kim, 2021) as well as bacterial cytochromes P450 (Kwon et al., 2013), cytochrome C oxidase (Katayama et al., 2010), and manganese peroxidase (Ninomiya et al., 2014). These examples all used *E. coli* lysate, which is by far the most commonly used CFPS system. *E. coli*-based CFPS is relatively inexpensive and scalable and has high productivity. However, it is only of limited use for the production of eukaryotic proteins for which post-translational modification (PTM) and correct folding might become a bottleneck. To address this problem, several eukaryotic CFPS systems, each having distinct features and thus qualifying them for special applications, were developed (Zemella et al., 2015). In this work, we focus on lysates containing endogenous microsomes. These microsomes are ER-derived vesicles that allow protein translocation and were reported for Chinese hamster ovary (CHO) (Brödel et al., 2015) and *Sf*21-based systems (Tarui et al., 2001). Microsomes enable posttranslational modifications such as N-glycosylations (Gurramkonda et al., 2018; Zemella et al., 2018), phosphorylation (Quast et al., 2016), lipidation (Shaklee et al., 2010), and signal peptide cleavage (Zemella et al., 2018). Furthermore, the microsomal lumen is much more favorable for the formation of disulfide bonds. This was shown by the example of antibody fragments synthesized in an insect-based CFPS reaction (Stech et al., 2014). Of those PTMs, N-glycosylation and disulfide bond formation are of particular interest when producing UPOs. Eukaryotic CFPS might thus prove itself a suitable platform for the synthesis of UPOs. This work aims to evaluate the capacity of the well-characterized *Sf*21 cell-free system to synthesize UPOs. The procedure for the cell-free protein production was shown for the model enzymes *Mro*UPO and *Aae*UPO as representative for short and long UPOs, respectively. We also sought to demonstrate the capability of this cell-free system through the synthesis of the novel short *Pan*UPO.

## 2 Materials and methods

### 2.1 Design of DNA templates

The templates for the *Sf*21-based synthesis of *Aae*UPO and *Mro*UPO were manufactured by BioCat GmbH (Heidelberg, Germany). The construct design was based on the design reported earlier (Brödel et al., 2013b). The constructs were equipped with a T7 promotor. An internal ribosome entry site (IRES) from the Cricket-paralysis virus was used to enhance cap-independent translation initiation. The native signal peptide (and propeptide in case of *Aae*UPO) was removed, and constructs with and without melittin signal peptide were prepared. The mature proteins of *Aae*UPO and *Mro*UPO were identified based on previous experimental data (Pecyna et al., 2009; Gröbe et al., 2011). *Pan*UPO propeptide was identified based on homology. The last codon of the melittin signal peptide coding for the C-terminal Asp was omitted. pUC57-1.8 k was used as a plasmid backbone.

### 2.2 Cell-free proteins synthesis

*Sf*21 lysate preparation and coupled CFPS have already been described and explained (Brödel et al., 2013b; Quast et al., 2015). Minor changes were made during lysate preparation. In all buffers used during the lysate preparation, DTT was omitted, and 0.05 mM GSH and 0.25 mM GSSG were added. Coupled transcription-translation reactions were performed in a batch mode format. Protein synthesis was performed in a thermomixer (Thermomixer comfort, Eppendorf, Hamburg, Germany) at 21°C (if not stated otherwise) and with gentle shaking at 500 rpm for 3 h. Reaction volumes of 50 μL were composed of 40% (v/v) *Sf*21 cell lysate, 100 µM of each canonical amino acid, nucleoside triphosphates (1.75 mM ATP, 0.30 mM CTP, 0.30 mM GTP, and 0.30 mM UTP), 100 ng/μL vector DNA, and 1 U/µL T7 RNA-polymerase (Agilent, Waldbronn, Germany), 0–75 µM porcine hemin (Alfa Aesar, Haverhill, USA) dissolved in NaOH. To monitor protein quality and quantity, reaction mixtures were supplemented with ^14^C-labeled leucine (Perkin Elmer, Inc.; Baesweiler, Germany). No template controls (NTC) were prepared in the same way as the samples except that the DNA template was replaced by water.

### 2.3 Fractionation

After incubation, the translation mix (TM) was centrifuged at 16,000 × g and 4°C for 10 min. The resulting supernatant (SN1) was collected, and the microsome harboring pellet was resuspended in phosphate-buffered saline (PBS) containing 0.5% 3-[(3-cholamidopropyl)dimethylammonio]-1-propanesulfonate (CHAPS). The resuspended pellet was incubated at 21°C and 750 rpm for 45 min in order to release translocated proteins out of the microsomes. The samples were then centrifuged again, and the supernatant (SN2) was collected.

### 2.4 Quantitative analysis through liquid scintillation counting

Total protein yields of cell-free synthesized, ^14^C-labeled protein were determined by hot trichloroacetic acid (TCA) precipitation and subsequent liquid scintillation counting (Stech et al., 2012). In short, 3 µl of the fraction analyzed were mixed with 3 ml 10% TCA (Carl Roth GmbH & Co. KG; Karlsruhe, Germany) with 2% casein hydrolysate (Carl Roth GmbH & Co. KG; Karlsruhe, Germany) and incubated in a water bath at 80°C for 15 min followed by 30 min incubation on ice. The TCA solution was then transferred to membrane filters (VWR International GmbH, Darmstadt, Germany) using a vacuum filtration device (Hoefer, Inc., Holliston, USA). The filters were washed with 5% TCA and dried with acetone. The dry filters were transferred into scintillation vials (Sarstedt AG & Co KG) and incubated in 3 ml scintillation cocktail for 1 h. The samples were then measured using the Hidex 600 SL (Hidex; Turku, Finland). Protein yields were calculated based on the counted disintegrations per minute, the specific activity of ^14^C-leucine in the cell-free reaction, the molecular weight, and the number of leucines in the protein.

### 2.5 SDS-PAGE analysis

For qualitative analysis, 3 µl of the fraction analyzed was mixed with 47 µl water and precipitated in 150 µl cold acetone (Carl Roth GmbH & Co. KG; Karlsruhe, Germany) for at least 15 min. After centrifugation at 16,000 × g at 4°C for 10 min, the supernatant was removed, and the precipitate dried for 30 min at 45°C. The precipitate was resuspended in NuPage™ loading buffer (Fisher Scientific GmbH, Schwerte, Germany). Samples were incubated at 70°C for 10 min. Samples were loaded onto NuPage™ 10% Bis-Tris Gels (Fisher Scientific GmbH, Schwerte, Germany). The SeeBlue Pre-Stained marker (Fisher Scientific GmbH, Schwerte, Germany) was used as standard. Gels were run at 185 V for 35 min and then dried at 70°C (Unigeldryer 3545D, Uniequip, Planegg, Germany). The dry gels were placed on a phosphor screen (GE-Healthcare GmbH; Munich, Germany). After 1–2 days, the phosphor screen was scanned using an Amersham Typhoon RGB (GE-Healthcare GmbH; Munich, Germany) to visualize labeled proteins.

### 2.6 Deglycosylation assay

Protein N-glycosylation was investigated using PNGaseF (peptide N-glycosidase F, New England Biolabs GmbH, Frankfurt am Main, Germany) according to the manufacturer’s protocol. Proteins were synthesized in a cell-free manner in the presence of ^14^C-leucine. 3 µl of the protein sample were then used for the deglycosylation assay and precipitated in acetone for SDS-PAGE analysis as described above.

### 2.7 Spectrophotometric assays

The activity of UPO was analyzed through the conversion of 2,2′-azino-bis(3-ethylbenzothiazoline-6-sulfonic acid) (ABTS) (Merck KGaA, Darmstadt, Germany) to the ABTS radical cation (ε_418_ = 36,000 mM^−1^ cm^−1^), two molecules of 2,6-dimethoxyphenol (DMP) (Merck KGaA, Darmstadt, Germany) to coerulignone (ε_469_ = 49,600 mM^−1^ cm^−1^) and 5-nitro-1,3-benzodioxole (NBD) (Merck KGaA, Darmstadt, Darmstadt, Germany) to 4-nitrocatechol (ε_425_ = 9,700 mM^−1^ cm^−1^). For the activity assay, 5 μL–20 µL of sample were added to 85 μL–70 µL of assay buffer. The assay buffer compositions were as follows: **ABTS-Assay**: McIlvaine buffer pH 4.5, 0.3 mM ABTS, 10 mM GSSG; **DMP-Assay**: 0.1 M potassium phosphate buffer pH 6 (*Mro*UPO) or pH 7 (*Aae*UPO), 6 mM DMP; **NBD-Assay**: McIlvaine buffer pH 7, 0.5 mM NBD. The reactions were started by adding 10 µL of 0.03% H_2_O_2_. Extinction measurements were performed using a microplate reader (Mithras^2^ LB 943 Multimode Plate Reade, Berthold Technologies GmbH & Co. KG).

### 2.8 Conversion of pharmaceuticals and LC-MS analysis

The UPO activities towards the pharmaceuticals propranolol, diclofenac, clopidogrel, and desipramine were analyzed by LC-MS. For this purpose, 10 µl of UPO sample were incubated with a reaction mixture containing 1 mM substrate, 1 mM hydrogen peroxide, 5 mM ascorbate, and 20 mM potassium phosphate buffer (pH 7) at 25°C and 800 rpm on a thermal shaker (TurboShaker 3,500, Scienova GmbH, Jena, Germany) for 5 min and stopped by adding 100 µl of cold acetonitrile (−20°C). The samples were centrifuged at 14,000 × g for 10 min, and the supernatants were analyzed by LC-MS. Calibrations were performed with authentic standards 4-hydroxypropranolol, 4′-hydroxydiclofenac, 2-oxoclopidogrel, and 2-hydroxydesipramine.

Chromatographic separations in LC-MS experiments were performed on a Thermo Scientific Vanquish Flex Quaternary UHPLC system (Fisher Scientific GmbH, Waltham, MA, USA) using a Kinetex^®^ column (C18, 2.6 µm, 100 Å, 150 × 2.1 mm, Phenomenex, Torrance, CA, USA). The injection volume was 1 μL, and the column was eluted at a flow rate of 0.5 ml min^−1^ and 40°C with two mobile phases: A (diH_2_O, 0.1% formic acid) and B (acetonitrile, 0.1% formic acid) and the following gradient: 0 min, 10% B; 0.5 min, 10% B; 5 min, 80% B; 7 min, 80% B; 7.1 min, 10% B; 10 min, 10% B. MS spectra were obtained using the Thermo Scientific Q Exactive Focus quadrupole-Orbitrap mass spectrometer (Thermo Electron, Waltham, MA, USA) coupled with a heated electrospray ionization source in positive mode. The tune operating parameters were as follows: rate of sheath gas flow and auxiliary gas flow—40 and 15 (arbitrary units), respectively; spray voltage—4.0 kV; the temperature of capillary and sample heater—260°C and 400°C, respectively; high-resolution MS was operated at full scan positive mode with a mass range of m/z 150–1,500 at a resolution of 70,000 (m/z 200).

## 3 Results

### 3.1 Setting up a cell-free reaction for the synthesis of unspecific peroxygenases

**FIGURE 1:**
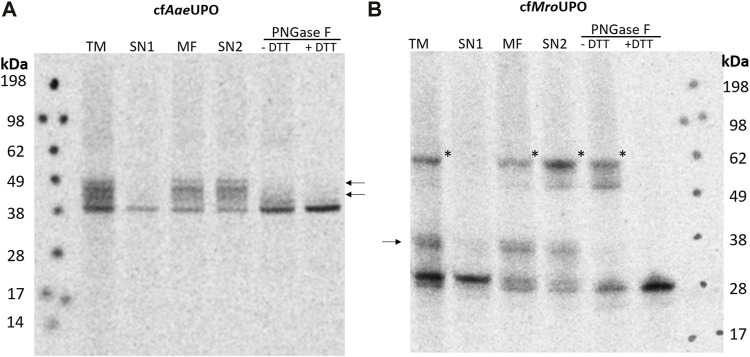
SDS-PAGE and autoradiography of cf*Aae*UPO and cf*Mro*UPO. Samples of the translation mix (TM), supernatant 1 (SN1, containing non-translocated protein), the microsomal fraction (MF), and supernatant 2 (SN2, containing translocated protein recovered from the microsomes) were acetone precipitated, separated by SDS-PAGE, and analyzed through autoradiography. Two additional SN2 samples were collected and subjected to PNGase F digestion. The PNGase F digestion was performed under both native and denaturing/reducing conditions. All samples were treated with non-reducing denaturing sample buffer and heated before performing SDS-PAGE. The analysis was performed for cf*Aae*UPO **(A)** and cf*Mro*UPO **(B)**. N-glycosylation is indicated by arrows, a possible dimer band is labeled by asterisks.

In order to obtain a universally applicable platform for UPO production, said platform must support glycosylation and disulfide bond formation. A *Sf*21-based lysate is thus used for the cell-free synthesis because of its high translocation and glycosylation efficiency (Batista et al., 2021). To achieve efficient translocation, the sequences coding for the native signal peptide and following residues that are processed during the maturation of *Aae*UPO and *Mro*UPO were replaced by the melittin signal peptide, which was shown to enhance translocation as well as translation efficiency in *Sf*21 and CHO CFPS (Brödel et al., 2013b; Brödel et al., 2014). The melittin signal peptide was modified by removing the C-terminal Asp, which is known to remain at the N-terminus of the processed target protein after signal peptide cleavage. The lysate was also supplemented with glutathione and glutathione-disulfide (GSH/GSSG) during lysate preparation in order to promote the formation of disulfide bonds. The translation mix (TM) was supplemented with ^14^C-leucine, thereby enabling visualization through autoradiography after SDS-PAGE as well as protein quantification. After synthesis, the TM was fractionated by centrifugation, resulting in supernatant 1 (SN1), which contains all soluble components of the cell-free reaction, and the pelleted microsomal fraction (MF), which contains the microsomes including the translocated protein. The microsomes were then treated with 0.5% CHAPS in order to release translocated protein from the microsomal lumen. Soluble proteins (SN2) were separated from any remaining insoluble debris by an additional centrifugation step. Using the redox optimized lysate, *Aae*UPO and *Mro*UPO were successfully synthesized (Figure 1A,B). For cell-free *Aae*UPO (cf*Aae*UPO), a band of about 38 kDa and for cf*Mro*UPO, a band of about 28 kDa was detected. This is well in accordance with the theoretical masses of *Aae*UPO and *Mro*UPO (38.4 kDa and 28 kDa, respectively). Also weaker bands of higher mass were observed for both UPOs in the TM, MF, and SN2; these bands are no longer present after PNGase F digestion, thereby indicating glycosylation. In the fractions TM, MF, and SN2 of cf*Mro*UPO a pronounced band of approx. 62 kDa was detected; which was no longer visible under reducing conditions. The band most likely corresponds to the expected homodimer.

**FIGURE 2:**
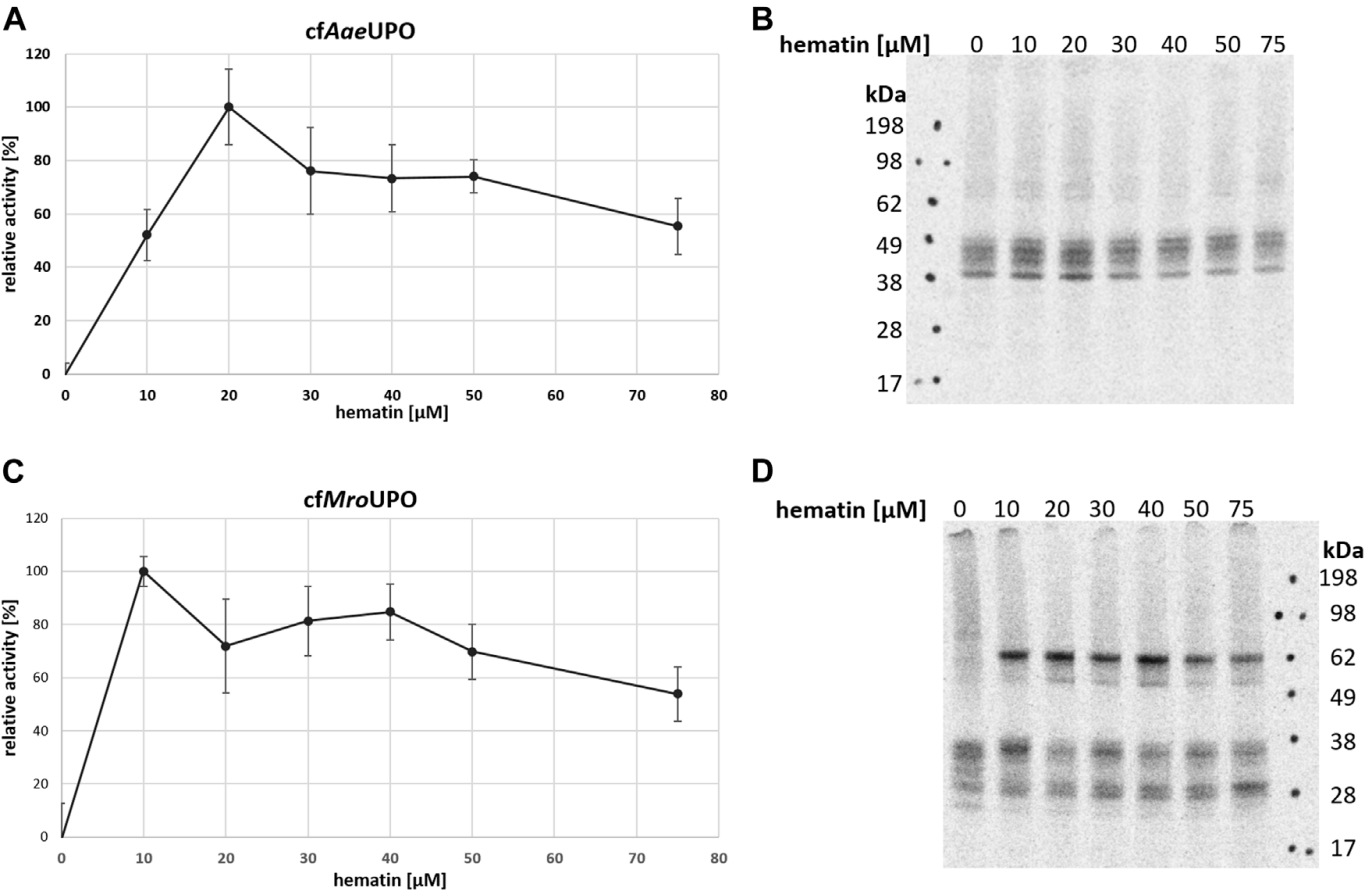
Influence of hematin supplementation on cfUPO activity. The activity of cf*Aae*UPO **(A)** and cf*Mro*UPO **(C)** when synthesized in the presence of differing amounts of hematin was determined by ABTS-Assay **(A)** or DMP-assay **(C)**. Measurements were performed in triplicate. For each sample, an NTC was analyzed and subtracted from the corresponding data points (B + D). SN2 of cf*Aae*UPO **(B)** and cf*Mro*UPO **(D)** synthesized at different hematin concentrations was separated by SDS-PAGE and visualized through autoradiography.

One major advantage of CFPS is the open nature of the system, which allows the easy manipulation of reaction parameters and buffer conditions. Many general factors such as temperature and salt and lysate concentrations influence both protein yields and activity. Insect-based CFPS has a wide adjustment range, which was sufficiently covered in previous studies (Tarui et al., 2001; Groll et al., 2007; Brödel et al., 2013b), thereby providing a framework for further reaction design. In order to obtain active enzyme, the reaction temperature was reduced from 27°C to 21°C (Supplementary Figure S1). To our knowledge, no hemoprotein had been produced in an insect-based CFPS until now. In order to assess the influence of heme addition on protein synthesis, different hematin concentrations were applied. To determine the enzyme activity, the conversions of ABTS to its radical cation and DMP to coerulignone were analyzed spectrophotometrically. For both UPOs, no activity was observed when hematin was not supplemented to the reaction. The optimum for cf*Aae*UPO and cf*Mro*UPO was 20 μM and 10 µM hematin, respectively (Figures 2A–C). Higher hematin concentrations decreased the UPO activity while increasing background activity. In order to account for background activity, a no-template-control (NTC) was analyzed for every sample. Data were corrected by the background activity. The autoradiograph of cf*Aae*UPO synthesized at different hematin concentrations showed no remarkable differences (Figure 2B). However, for cf*Mro*UPO, the formation of dimers is drastically reduced when hematin is omitted (Figure 2D).

### 3.2 Impact of translocation and signal peptide cleavage on UPO activity

**FIGURE 3:**
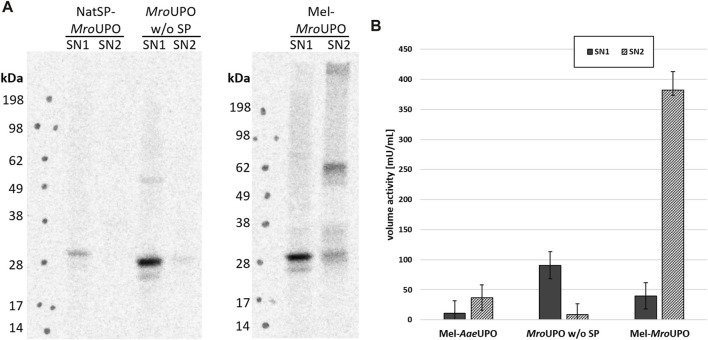
Influence of translocation on cfUPO activity. **(A)** Different *Mro*UPO constructs either without signal peptide (*Mro*UPO w/o SP), with native signal peptide (NatSP-*Mro*UPO), or with melittin signal peptide (Mel-*Mro*UPO) were synthesized, and supernatant 1 (SN1, containing non translocated protein) and supernatant 2 (SN2, containing translocated protein recovered from the microsomes) were analyzed by SDS-PAGE with subsequent autoradiography. **(B)** The conversion of DMP to coerulignone was analyzed spectrophotometrically for cf*Aae*UPO, cf*Mro*UPO without SP, and Mel-*Mro*UPO.

In order to further elucidate the contribution of the melittin signal peptide (Mel) and protein translocation to the activity of cell-free synthesized UPO, additional UPO templates were designed. These constructs contain either the complete amino acid sequence of the unprocessed wild-type UPOs (including the native signal peptide and, if present, further residues that are cleaved during maturation) or only the mature UPO. In the case of cf*Aae*UPO, CFPS was successful only when the melittin signal peptide was used (Supplementary Figure S2). In contrast, all three cf*Mro*UPO variants were successfully synthesized independently of any signal peptide. However, the cf*Mro*UPO without signal peptide showed only a low translation efficiency. Using the native signal peptide led to an increase of synthesized cf*Mro*UPO, that was located mainly in the SN1 and formed dimers with low efficiency. The introduction of a melittin signal peptide not only resulted in a higher overall protein yield but also led to translocated protein in the SN2 with glycosylation and dimerization as described above (Figure 3A).

The activity of Mel-cf*Aae*UPO as well as of cf*Mro*UPO with and without native signal peptide was analyzed by the conversion of DMP. By far, the highest volume activity of 382 mU/mL was observed for Mel-cf*Mro*UPO in the SN2 compared with 40 mU/mL in the SN1. Omitting the signal peptide leads to increased activity in the SN1 (91 mU/mL). However, no activity was displayed in the SN2 without signal peptide. The volume activity of Mel-cf*Aae*UPO in the SN2 was 37 mU/mL while no activity was observed in the SN1 of Mel-cf*Aae*UPO. Because the translocation seems to play a pivotal role in terms of UPO activity, all further experiments were performed with the melittin constructs. Background activity detected in the NTC was determined to be 14 mU/mL and 22 mU/mL in the SN1 and SN2, respectively. In order to account for this background, for every sample an NTC was analyzed for every sample (Figure 3B).

### 3.3 Evaluation of the activity of cfUPOs towards different substrates

ABTS was successfully converted by both UPOs obtained from SN2 (volume activities of 490 mU/mL and 29.5 mU/mL for cf*Aae*UPO and cf*Mro*UPO, respectively (Supplementary Figure S3A), which corresponds to specific activities of 314 U/mg and 18.5 U/mg (Table 1). Applying the SN1 sample to the ABTS-assay buffer led to clouding regardless of the presence of UPO in the sample. Therefore, no colorimetric data was obtained for the SN1 by means of the ABTS assay.

**TABLE 1:** Specific activities of cf*Aae*UPO and cf*Mro*UPO for different chromogenic substrates.

	Specific activity [U/mg]	
	cf*Aae*UPO	cf*Mro*UPO
DMP	27 ± 15.6	221 ± 10.3
ABTS	341 ± 48	18.5 ± 10.91
NBD	1.7 ± 0.22	1.4 ± 0.57

The reaction products in the ABTS and DMP assay are the result of a one-electron oxidation, which is a classical peroxidase reaction. While peroxidase activity is expected from UPOs (Ullrich et al., 2004), their true potential lies in their peroxygenase activity. Therefore, cf*Aae*UPO and cf*Mro*UPO were analyzed for the conversion of NBD to 4-nitrocatechol. The specific activities were 1.7 U/mg and 1.4 U/mg for cf*Aae*UPO and cf*Mro*UPO, respectively. The volume activities were 4.7 mU/mL and 5.2 mU/mL for cf*Aae*UPO and cf*Mro*UPO, respectively. This was more than 1.5-fold higher than the background activity of 2.8 mU/mL (Supplementary Figure S3B).

The conversion of chromogenic model substrates as shown above is a suitable tool for screening purposes. However, these substrates play a subordinate role when it comes to the application of UPOs. One of those applications is the biocatalytic synthesis of pharmaceuticals and their metabolites. In this work, we analyzed the conversion of four different pharmaceutically relevant substrates by LC-MS: *β*-adrenergic blocker propranolol to 5-hydroxypropanolol, anti-inflammatory diclofenac to 4′-hydroxydiclofenac, antithrombotic prodrug clopidogrel to 2-oxoclopidogrel, and antidepressant desipramine to its 2-hydroxy-derivate. cf*Aae*UPO showed volume activities in the mU/mL range for propranolol, diclofenac, and clopidogrel (Table 2). The conversion of desipramine by cf*Aae*UPO was also shown, albeit at much lower levels. In contrast, cf*Mro*UPO samples exhibited a noticeably lower volume activity towards propranolol, diclofenac, and desipramine, whereas the volume activity towards clopidogrel exceeds that of cf*Aae*UPO by more than 2-fold.

**TABLE 2:** Activity of cf*Aae*UPO and cf*Mro*UPO for the conversion of different pharmaceutically relevant molecules.

	Volume activity (µU/mL)		
	cf*Aae*UPO	cf*Mro*UPO	NTC
Propranolol	3,897 ± 308	169 ± 2	8 ± 2
Diclofenac	6,159 ± 100	607 ± 14	136 ± 6
Clopidogrel	3,412 ± 13	8,803 ± 266	9 ± 2
Desipramine	146 ± 3	56 ± 3	35 ± 1

### 3.4 Cell-free synthesis of novel *Podospora anserina* UPO

**FIGURE 4:**
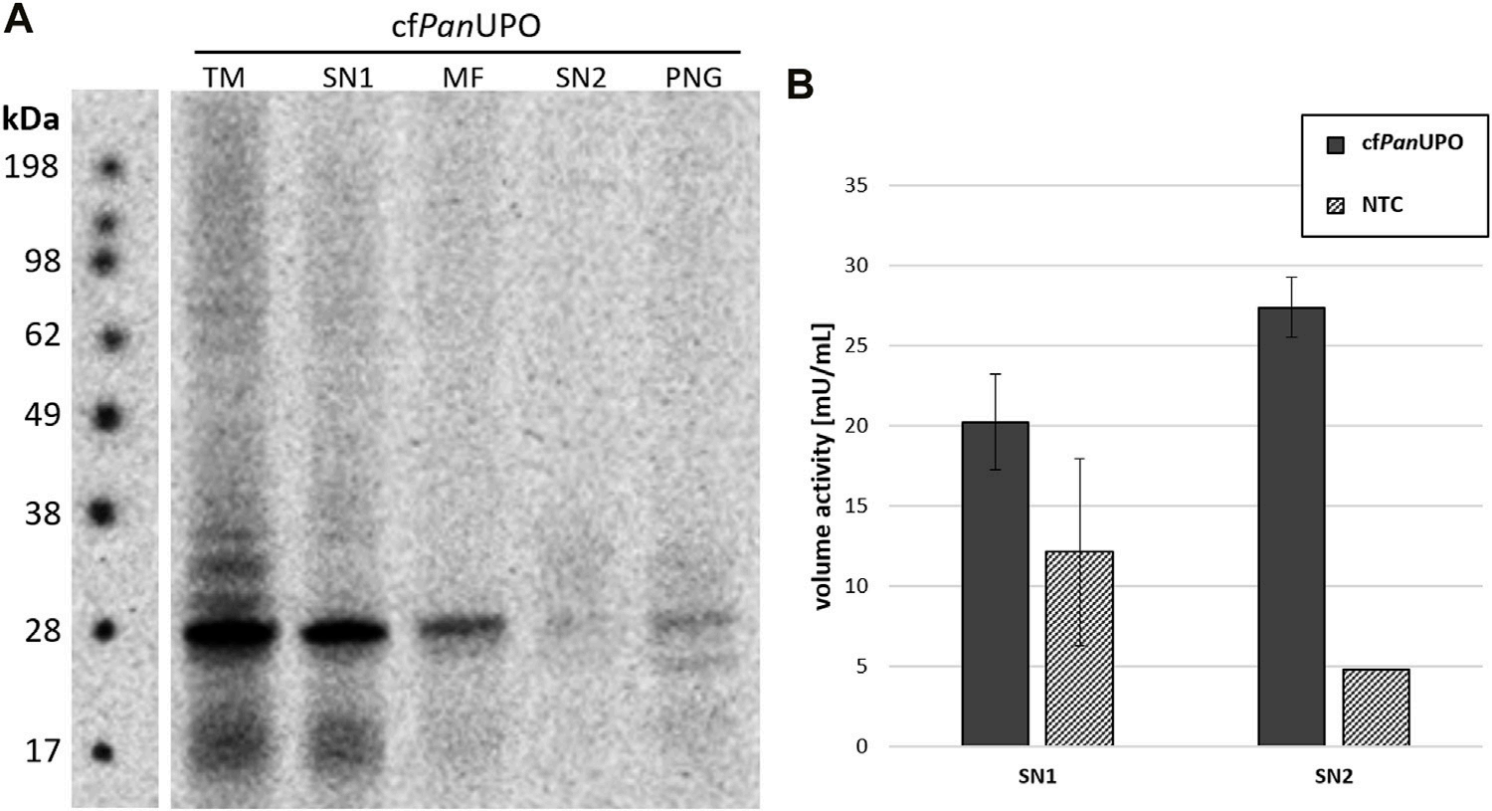
Cell-free synthesis of novel *Pan*UPO. **(A)** SDS-PAGE and autoradiography of cf*Pan*UPO. Samples of the translation mix (TM), supernatant 1 (SN1, containing non-translocated protein), the microsomal fraction (MF), and supernatant 2 (SN2, containing translocated protein recovered from the microsomes) were acetone precipitated, separated by SDS-PAGE, and analyzed by autoradiography. An additional SN2 sample was subjected to a native PNGase F digestion (PNG). All samples were treated with non-reducing denaturing sample buffer and heated prior to SDS-PAGE **(B)** DMP conversion of cf*Pan*UPO from SN1 and SN2 compared with the background activity in those fractions.

We then sought to expand the scope of available UPOs with our new cell-free system. To do so, we prepared a template containing a putative UPO sequence from the filamentous ascomycete *Podospora anserina.* As for the other UPOs, the signal peptide was removed and exchanged for a modified melittin signal peptide as described above. The SDS-PAGE with subsequent autoradiography showed a band of about 28 kDa in all fractions; this is well in accordance with the theoretical size of 29.9 kDa (Figure 4A). Additional bands above the main band were observed in the TM fraction. In SN2, only a faint smear was present; which becomes visible as a distinct band after PNGase F digestion, thereby indicating heterogenic glycosylation. Subjecting SN1 and SN2 of cf*Pan*UPO to the DMP assay revealed activity of 27.4 mU/mL in SN2 (Figure 4B). The *Pan*UPOs conversion of the four pharmaceutical molecules investigated for *Aae*UPO and *Mro*UPO was investigated through LC-MS. Considerable activity was observed only for clopidogrel (Table 3).

**TABLE 3:** Activity of cf*Pan*UPO for the conversion of different pharmaceutically relevant molecules.

	Volume activity (µU/mL)		
	cf*Pan*UPO	NTC
Propranolol	69 ± 308	25 ± 4
Diclofenac	280 ± 100	258 ± 37
Clopidogrel	1757 ± 13	12 ± 9
Desipramine	54 ± 3	17 ± 3

## 4 Discussion

Because of their broad catalytic spectrum, UPOs are highly attractive biocatalysts, especially for the generation of molecules of pharmaceutical relevance. For example, *Mro*UPO is capable of cleaving corticosteroid side chains and can thus provide intermediates for the industrial synthesis of steroids (Ullrich et al., 2018). Another example would be the conversion of the anticancer drug cyclophosphamide using UPOs in order to circumvent the complex and inefficient chemical synthesis of its drug metabolite. The efficiency of the conversion of cyclophosphamide was dependent on the UPO that was used (Steinbrecht et al., 2020). Substantial differences in the catalytic activity of different UPOs were also reported for substrates like long chain terminal alkenes (Babot et al., 2022) and isophorone derivatives (Aranda et al., 2019). This underlines the need for a comprehensive UPO toolbox to take advantage of the full catalytic potential of UPOs. In order to expand this toolbox, we sought to establish a vesicle-based CFPS approach for the synthesis of long and short UPOs. We first aimed to produce the well-characterized UPOs *Aae*UPO and *Mro*UPO because the data available on the activity of these UPOs would facilitate the evaluation of the performance of the *Sf*21-based cell-free system. In this work, we have shown that both *Aae*UPO and *Mro*UPO were successfully synthesized in an *Sf*21-based cell-free reaction. For both UPOs, glycosylation was confirmed through PNGase F digestion. As expected, glycosylation was not observed in SN1 because it requires translocation into the microsomes. A downshift in the protein size of cf*Mro*UPO from SN1 to the MF indicates the successful cleavage of the melittin signal peptide during the translocation. A similar slight downshift can also be observed for *Aae*UPO; however, this shift is much more subtle because of the resolution of the SDS-PAGE at higher molecular weights. Because the N-terminus of *Aae*UPO is rather sensitive and requires accurate processing (Püllmann et al., 2021), its activity supports the assumption of a correctly cleaved signal peptide. Both N-glycosylation and signal peptide cleavage has been reported for *Sf*21-based CFPS (Zemella et al., 2018). UPOs are N-glycosylated proteins, and studies on *Aae*UPO showed that their glycosylations are of the high mannose type (Pecyna et al., 2009; Piontek et al., 2010; Piontek et al., 2013). High mannose type glycosylation can also be expected for *Sf*21 CFPS (Zemella et al., 2018); however, the glycan structure might be less complex. The presence of multiple bands or smears of glycosylated protein indicates non-homogenous glycosylation, whereby not all glycosylation sites are addressed and/or are differently glycosylated (Zemella et al., 2018). Different glycoforms of *Aae*UPO and *Mro*UPO are also observed when they are expressed in their wild-type host; however, the differences in glycosylation appear to be more subtle (Ullrich et al., 2009; Gröbe et al., 2011). In addition to the glycosylation, cf*Mro*UPO displayed another band at about 62 kDa, which indicates dimer formation. This band was no longer detectable after applying reducing conditions during the deglycosylation assay. This is in accordance with the reported 3D structure of *Mro*UPO (PDB 5FUJ), which forms dimers through intermolecular disulfide bridges. Although these results indicate dimerization through disulfide bonds, a definite proof (e.g., through mass spectrometry) is necessary. These potential dimers were predominantly observed in the SN2; this is not surprising because disulfide bonds are not expected to form in a cytosolic environment like the SN1. However, the possible presence of dimers in the SN1 could be a result of the redox optimization that was performed on the lysate used. Previous studies applied redox optimizations with varying results. While the redox supplementation of an insect-based cell-free reaction barely increased the activity of antibody fragments (Stech et al., 2014), the addition of not only GSH/GSSG but also protein disulfide isomerase to an insect-based cell-free reaction resulted in disulfide bridged alkaline phosphatase and human lysozyme (Ezure et al., 2007). A third approach for redox-optimization was utilized in this work; GSH/GSSG were added during lysate preparation, thereby resulting in a ready-to-use redox-optimized CFPS system that requires no further supplementation. A similarly optimized lysate was shown to promote the activity of ice structuring proteins (Brödel et al., 2013a); however, the reaction mix was also supplemented with GSH/GSSG in this case.

To the best of our knowledge, this is the first time a hemoprotein was synthesized in an insect-based CFPS reaction. We thus analyzed the effect of hematin on the cell-free synthesis and UPO activity. For that, two different assays (ABTS and DMP) were chosen to maximize the readout because both UPOs have differing activities towards these substrates (Ullrich et al., 2004; Gröbe et al., 2011). For both UPOs, no activity was observed without hematin supplementation, thereby indicating that no or at least an insufficient amount of heme is present in the unsupplemented lysate. Without the addition of hematin, no dimer formation of *Mro*UPO was observed, thus indicating insufficient folding in the absence of heme. Increasing UPO activity was observed with rising hematin concentrations up to 10 μM–20 µM. No remarkable effect of the hematin or its solvent on the protein amount in SN2 was observed. For the synthesis of other hemoproteins in *E. coli* lysate, where heme was supplemented as hemin, an optimal hemin concentration in the range of 0.15 µM (Katayama et al., 2010) to 16.7 µM (Zhu et al., 2015) was reported. As an alternative to heme supplementation, the heme can be generated *in situ* from glucose and glycine in the presence of 5-aminolevulinic acid synthase as shown for bacterial CYP (Kwon et al., 2013) and HRP (Park and Kim, 2021). However, because hemin is inexpensive and seems to have no major adverse effects towards the CFPS itself, there currently is no need for following that approach in the *Sf*21-based CFPS system.

The contribution of the signal peptide and translocation to the UPO activity was then assessed. The introduction of the melittin signal peptide had a drastic effect on translation. Whereas no cf*Aae*UPO was synthesized without Mel, the cf*Mro*UPO was synthesized independently of the use of the melittin signal peptide. However, the melittin signal peptide increased overall yields of cf*Mro*UPO. This is well in accordance with studies that found a similar positive effect of melittin on translation efficiency (Brödel et al., 2013a; Stech et al., 2014). The conversion of DMP by cf*Mro*UPO with and without melittin signal sequence was analyzed. cf*Mro*UPO without melittin signal sequence showed activity only in the SN1. The activity observed was even higher than for Mel-cf*Mro*UPO even though Mel-cf*Mro*UPO had similar protein yields in SN1. This reduced activity results from the uncleaved signal peptide at the N-terminus, which is close to the active center of the protein. Deviations from the native N-terminus of the mature UPO were shown to have a negative effect on activity (Püllmann et al., 2021). The SN2 of Mel-cf*Mro*UPO showed the highest activity by far. This underlines the importance of translocation for protein activity. The effect of the translocation on the enzyme activity can presumably be traced back to three factors: the formation of disulfide bonds, N-glycosylation, and protein folding in general. While analysis of the latter is difficult, the contribution of the other factors is more easily to assess. So far, there have been no studies of those factors on the activity of cell-free synthesized UPO. However, the effect of PTMs varies from UPO to UPO. For example, not all UPOs form disulfide bonds (Hofrichter et al., 2015), while glycosylations are present in all known UPOs; however, their impact on protein activity differs greatly depending on the individual UPO. *Aae*UPO was barely affected by deglycosylation, whereas *Mro*UPO showed considerably lower activity when deglycosylated. Some UPOs activity is completely abolished upon deglycosylation (Püllmann et al., 2021). When further evaluating the contribution of PTMs to the activity of cfUPO, these factors have to be considered.

As expected, cf*Mro*UPO exhibits a higher activity towards DMP than *Aae*UPO (Ullrich et al., 2004; Gröbe et al., 2011). For the substrate ABTS, the specific activity is in the range of wild-type *Aae*UPO (wt*Aae*UPO) with 295.7 U/mg (Ullrich et al., 2004), while the expression of an *Aae*UPO variant with an optimized signal peptide for *P. pastoris* expression achieved a specific activity of 233 U/mg (Molina-Espeja et al., 2014). When cfUPOs were compared with conventional prepared UPOs, the activity assays for the cfUPOs were performed using UPO directly after fractionation without any further purification. The cell-free synthesis of *Aae*UPO in a fungal lysate was recently shown (Schramm et al., 2022). The comparison shows that the activities of the cf*Aae*UPOs from both systems are in a comparable range. However, some different trends must be highlighted. While we achieved a higher volume activity of *Sf*21 cf*Aae*UPO towards ABTS, the cf*Aae*UPO synthesized in a fungal system shows a higher activity towards NBD. The conversion of pharmaceutical molecules also shows divergent trends. The fungal cf*Aae*UPO showed by far the highest activity towards propranolol; this was even higher than the wt*Aae*UPO. However, the *Sf*21 cf*Aae*UPO showed the highest activity towards diclofenac followed by propranolol and clopidogrel with about half the volume activity. These different catalytic profiles are surprising considering that they are both variants of the same UPO. Possible explanations include the presence of a His-tag at the C-terminus of the cf*Aae*UPO produced in fungal lysate, or differences in the glycosylation pattern and protein folding because of the presence of chaperones. In addition, buffer conditions such as salt concentrations and reaction components could affect UPO activity, especially in the cell-free approaches in which unpurified UPO was used for activity studies. Further investigation will be necessary to investigate the influence of these different parameters on catalytic activity. Taking advantage of these factors could provide a valuable approach to fine-tuning UPO activity. The data obtained analyzing the activity of both UPOs showed that the detection of UPO activity is tightly linked to the substrate applied. This underlines, that in pursuit of novel UPOs not only a versatile synthesis/expression platform but also a versatile analytical approach for testing different substrates is necessary. A promising step in this direction is the MISER (multiple injections in a single experimental run) approach (Knorrscheidt et al., 2020; Knorrscheidt et al., 2021), which allowed the analysis of multiple substrates and products at the same time in order to identify improved variants of the recently described *Myceliophthora thermophila* UPO (*Mth*UPO) (Knorrscheidt et al., 2021). For the successful analysis of a high amount of UPOs, production strategy and analysis have to be well matched. As an easy-to-handle and versatile platform, CFPS is highly compatible and can support high-throughput approaches.

The true potential of CFPS lies in its flexibility. Yield-wise, CFPS will hardly compete with conventional expression strategies on an industrial scale. Yields reported for *Mro*UPO produced in its wild-type host reach up to about 450 mg/L (Gröbe et al., 2011). While the expression of *Aae*UPO in its wild-type host yields only 10–30 mg/L (Ullrich et al., 2004; Pecyna et al., 2009), the recombinant expression of an *Aae*UPO variant, improved for secretion, yields over 200 mg/L (Molina-Espeja et al., 2015) with a recently reported pilot-scale production approach yielding 290 mg/L in a 2500 L fermentation (Tonin et al., 2021). The CFPS reaction shown here produced 1–2 mg/L of cfUPO. However, the scale of CFPS was much smaller. Reaction volumes typically are below 1 ml. Although 100 L CFPS reactions have been reported for *E. coli* CFPS (Zawada et al., 2011), we do not aim to present industrial-scale production levels. In this study, we sought to scale down the production to in order to achieve a high-throughput screening system. The downscaling of screening processes is an elementary feature of CFPS, which, on a larger scale, normally tends to have a higher cost per synthesized protein than common recombinant expression approaches (Thaore et al., 2020). While a higher price is acceptable for pharmaceutical applications (e.g., personalized medicine), the examples for industrial enzymes are less abundant and rely mainly on *E. coli* CFPS (Boyer et al., 2008; Li et al., 2016). Here, we sought for a fast and flexible screening platform in order to speed up engineering and functional characterization. Cell lysates and other components can be prepared in batches and stored over long periods. The CFPS reaction can then be prepared on demand. Depending on the assay applied, results can be obtained only 5 h after the reaction was started, thus allowing increased flexibility and handling. The capability of cell-free systems for those screening approaches was recently demonstrated by the synthesis and characterization of several novel azoreductases (Rolf et al., 2022).

We have shown that insect-based CFPS can be used to generate a novel short UPO from *Podospora anserina*. Initial activity assessment through DMP conversion revealed a lower activity of *Pan*UPO compared with *Aae*UPO and *Mro*UPO. Subsequent LC-MS analysis of the conversion of pharmaceutical substrates showed considerable activity only towards clopidogrel. This catalytic profile similar to that of *Mro*UPO coincides with a previously reported tendency observed for short UPOs to catalyze the hydroxylation of benzene rings and related structures with lower efficiency than long UPOs (Hofrichter et al., 2022).

The example of *Pan*UPO shows that the synthesis platform described is not only able to provide short and long UPOs but is also a promising approach to expanding the scope of available UPOs. As pointed out, some UPOs are still difficult to express in cell-based systems. The success depends not only on the expression itself but also on secretion and folding (Püllmann et al., 2021). CFPS might allow the decoupling of those factors. The secretion (or in this case translocation in the microsomes) uses a melittin signal peptide that leaves the N-terminus unaltered after cleavage. This approach worked well for *Aae*UPO, which was described to be quite sensitive to different signal peptides investigated for expression in yeast (Püllmann et al., 2021). An improvement in folding might be achieved by the supplementation of chaperones. The addition of eukaryotic chaperones to *E. coli*-based cell-free systems for the production of eukaryotic target proteins has been shown to have a beneficial effect on protein folding (Bonomo et al., 2010). Another approach to increase the activity of cell-free produced UPOs might be the use of a fungal system. This was recently shown for a novel fungal lysate (Schramm et al., 2022). While the use of fungal CFPS might be favorable because of its phylogenetic proximity to UPO producers and both host systems used do, in fact, produce UPOs themselves, there is yet much work to be put into further optimizing these lysates in order to harness their full potential. Because insect-based CFPS is well established, there are several methods available to address bottlenecks in protein yield and folding. These include methods to increase protein yields (e.g., dialysis-based approaches; (Quast et al., 2016); as well as repetitive synthesis approaches that can be used to increase the amount of translocated protein (Sachse et al., 2013). Another advantage is the flexible use of different templates. The mentioned fungal CFPS of UPOs so far relies on mRNA as a template. This requires *in vitro* transcription and purification of the mRNA before the translation reaction (Schramm et al., 2022). While decoupling transcription and translation reactions can have advantages, it is still cumbersome and hard to integrate into streamlined screening processes. Insect-based CFPS can be performed from either an RNA (Tarui et al., 2001) or a DNA template. Beyond that, the DNA templates can be either plasmids or linear DNA fragments (Ramm et al., 2022). Both approaches also apply to UPOs, thereby giving the insect-based CFPS higher flexibility for screening approaches. Overall, this makes insect-based CFPS a sophisticated screening platform for the identification and characterization of novel UPOs and variants thereof.

We successfully demonstrated that insect-based CFPS is well suited for the production of long and short UPOs using the example of the model enzymes *Mro*UPO and *Aae*UPO as well as the novel *Pan*UPO. The synthesis of active UPO was possible using translocationally active microsomes in the insect lysate. This work clearly depicts the importance of such vesicles and their capability to perform PTMs in order to obtain UPO. The open nature of CFPS in general and the well-established technologies for insect-based CFPS in particular make this platform a promising candidate for comprehensive UPO screening in order to identify novel UPOs and thus expand the biocatalytic toolbox for the production of pharmaceuticals and fine chemicals.

## Data Availability

The raw data supporting the conclusions of this article will be made available by the authors, without undue reservation.

## Abbreviations

*Aae*UPO, *Agrocybe aegerita* UPO; ABTS, 2,2′-azino-bis(3-ethylbenzothiazoline-6-sulfonic acid; CFPS, cell-free protein synthesis; cfUPO, cell-free synthesized UPO; DMP, 2,6-dimethoxyphenol; Mel, melittin signal peptide; MF, microsomal fraction; *Mro*UPO, *Marasmius rotula* UPO; NBD, 5-nitro-1,3-benzodioxole; *Pan*UPO, *Podospora anserina* UPO; PTM, post-translational modification; SN1, supernatant 1; SN2, supernatant 2; TM, translation mix.
