# Supplementary material for: Vesicle-based cell-free synthesis of short and long unspecific peroxygenases
Source: Front Bioeng Biotechnol. 2022 Nov 1;10:964396. doi: 10.3389/fbioe.2022.964396 (PMC9663805; doi:10.3389/fbioe.2022.964396)
Supplement: Supplementary file 1 [file DataSheet1.docx]

Supplementary Material


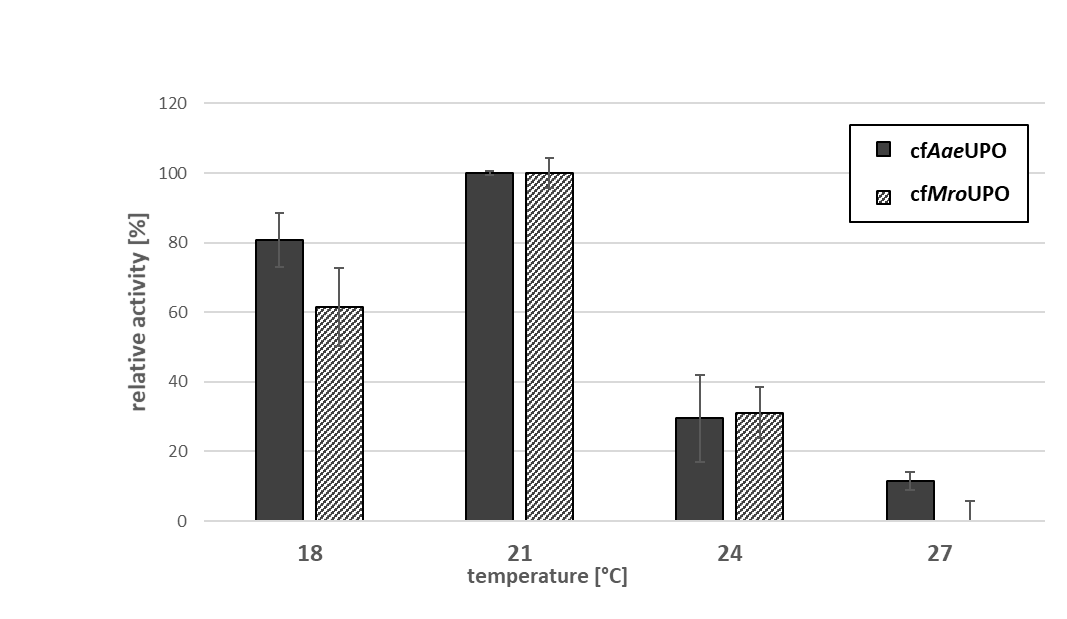


Figure S 1: Activity of cf*Aae*UPO and cf*Mro*UPO synthesized at different temperatures. The activities for the conversion of ABTS (*Aae*UPO) and DMP (*Mro*UPO) were determined in triplicate measurements. The activities are given as relative activities.

*
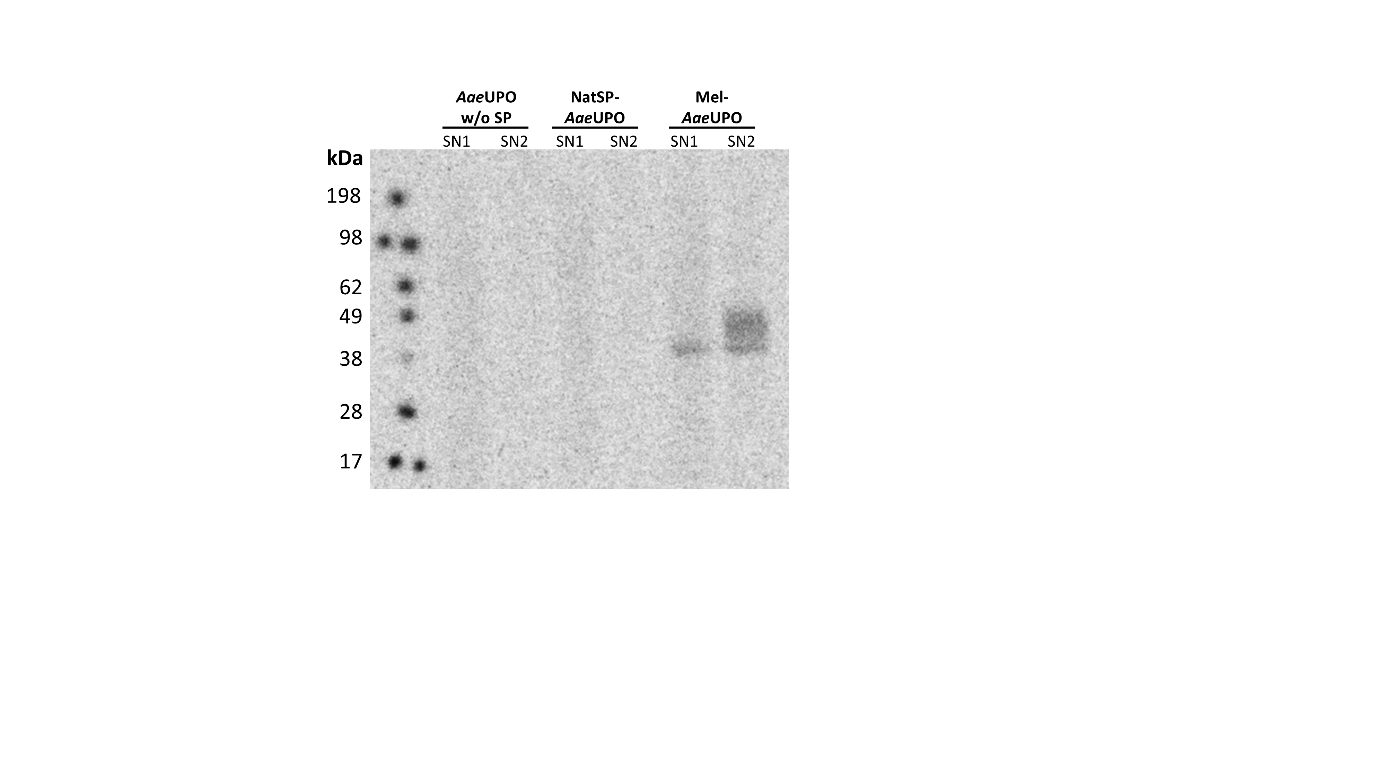
*

Figure S 2: Different *Aae*UPO constructs either without signal peptide (*Aae*UPO), with native signal peptide (NatSP-*Aae*UPO) or with melittin signal peptide (Mel-*Aae*UPO) were synthesized and the fractions SN1 and SN2 were analyzed by SDS-PAGE with subsequent autoradiography.

*
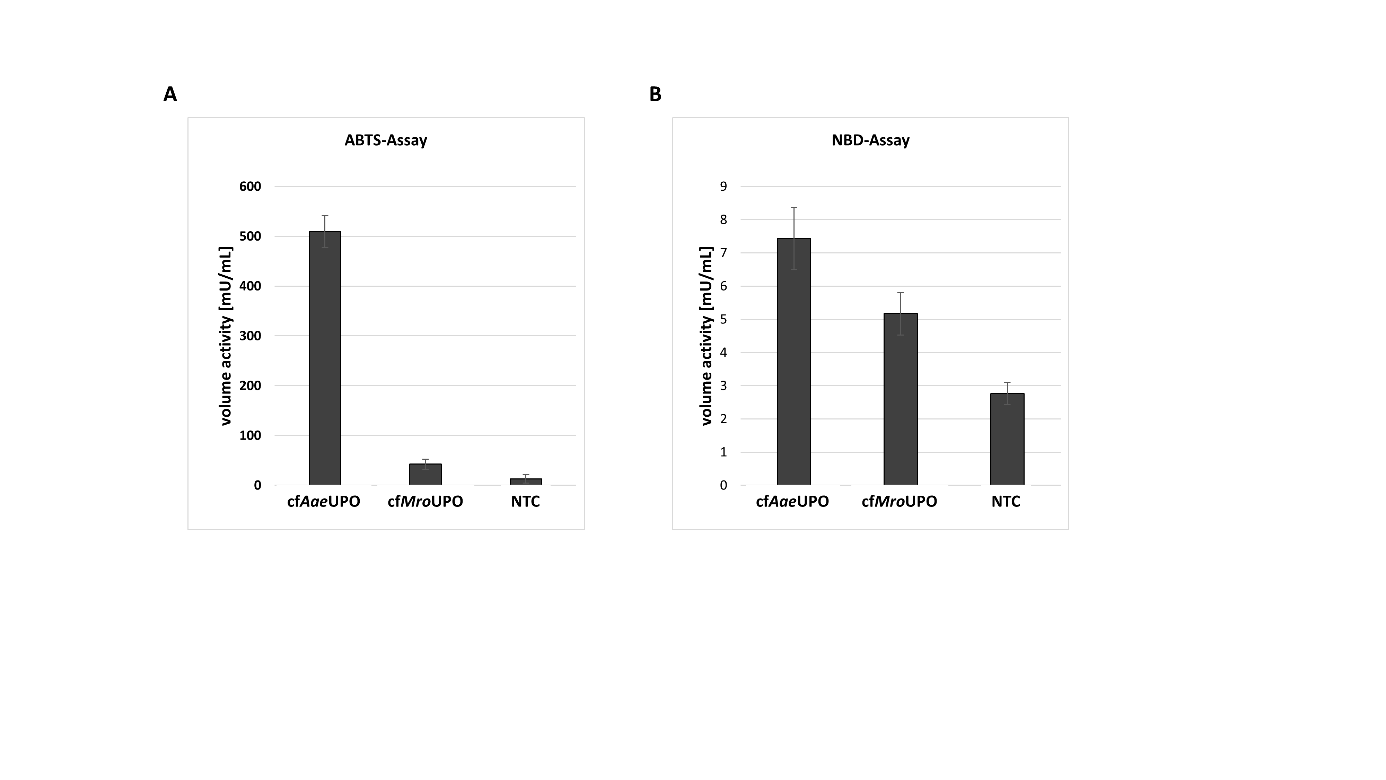
*

Figure S 3: Activity of cf*Aae*UPO and cf*Mro*UPO determined by conversion of ABTS (A) and NBD (B), respectively. Activities were determined by triplicate measurements. Additionally, a no template control (NTC) was performed to account for background activity.
